# Serine-ubiquitination regulates Golgi morphology and the secretory pathway upon *Legionella* infection

**DOI:** 10.1038/s41418-021-00830-y

**Published:** 2021-07-20

**Authors:** Yaobin Liu, Rukmini Mukherjee, Florian Bonn, Thomas Colby, Ivan Matic, Marius Glogger, Mike Heilemann, Ivan Dikic

**Affiliations:** 1 Institute of Biochemistry II, School of Medicine, Goethe University Frankfurt, Frankfurt am Main, Germany; 2 Buchmann Institute for Molecular Life Sciences, Goethe University Frankfurt, Frankfurt am Main, Germany; 3 Max Planck Institute for Biology of Ageing, Cologne, Germany; 4 CECAD Cluster of Excellence, University of Cologne, Cologne, Germany; 5 Institute for Physical and Theoretical Chemistry, Goethe-University Frankfurt, Frankfurt am Main, Germany

**Keywords:** Biochemistry, Cell biology

## Abstract

SidE family of *Legionella* effectors catalyze non-canonical phosphoribosyl-linked ubiquitination (PR-ubiquitination) of host proteins during bacterial infection. SdeA localizes predominantly to ER and partially to the Golgi apparatus, and mediates serine ubiquitination of multiple ER and Golgi proteins. Here we show that SdeA causes disruption of Golgi integrity due to its ubiquitin ligase activity. The Golgi linking proteins GRASP55 and GRASP65 are PR-ubiquitinated on multiple serine residues, thus preventing their ability to cluster and form oligomeric structures. In addition, we found that the functional consequence of Golgi disruption is not linked to the recruitment of Golgi membranes to the growing *Legionella*-containing vacuoles. Instead, it affects the host secretory pathway. Taken together, our study sheds light on the Golgi manipulation strategy by which *Legionella* hijacks the secretory pathway and promotes bacterial infection.

## Introduction

Ubiquitination is a post-translational modification that is conserved from yeast to mammals. The catalysis of canonical ubiquitination is regulated via a well-known E1-E2-E3 three-enzyme cascade in an ATP dependent manner [1]. Protein ubiquitination virtually regulates every cellular processes, including protein stability, protein trafficking, immunity, and DNA repair [2–5].

Consistent with the critical roles of ubiquitination in cellular processes, emerging evidence indicates that pathogens hijack the ubiquitination machinery for efficient invasion [6–8]. Various studies have revealed that effectors of the SidE family (SdeA, SdeB, SdeC and SidE) catalyze an NAD^+^-dependent, ATP-independent type of ubiquitination without the need of E2 and E3 enzymes [9, 10]. Moreover, unlike the conventional ubiquitination that occurs on lysine residues of substrate proteins, SidE family effectors catalyze the conjugation of Ub via a phosphoribosyl moiety to serine residues of host substrate proteins by a two-domain catalytic relay: a mono ADP-ribosyl transferase (mART) domain and a phosphodiesterase (PDE) domain [11–14].

Phosphoribosyl (PR)-linked ubiquitination can be reversed by DupA, a deubiquitinase with specific affinity for PR-ubiquitinated substrates [15]. In our previous study, we used the catalytically mutant DupA as a bait to identify targets of SdeA. Besides ER-related substrates, we also identified proteins related to other cellular pathways, including Golgi proteins, mitochondrial proteins and components of the autophagy machinery [15]. However, the biological functions of PR-ubiquitination of these proteins remained unclear. In this study, we made use of biochemical and microbiological approaches to characterize the PR-ubiquitination of Golgi tethering proteins GRASP55 and GRASP65 by SdeA. We also provide explanations for the Golgi morphological regulation by the PR-ubiquitination of these proteins. Moreover, we demonstrate that PR-ubiquitination regulates the host cellular secretory pathway during bacterial infection.

## Results

### SdeA is targeted to the ER and Golgi via its carboxyl terminus

**Fig. 1:**
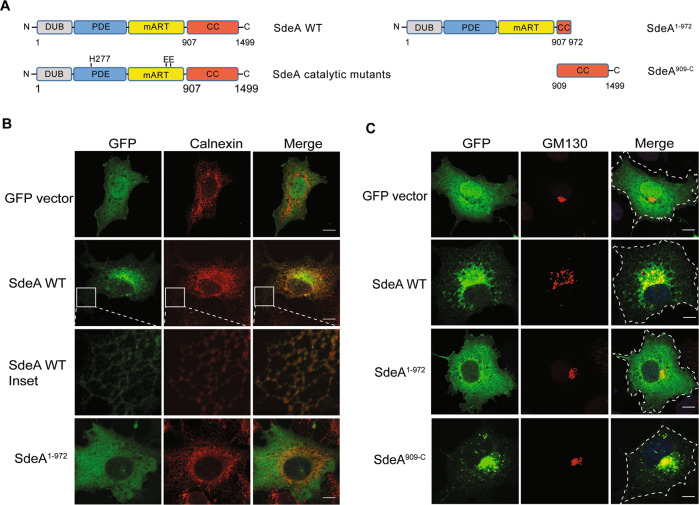
SdeA partially localizes to the Golgi. **A** Schematic diagrams of full-length wild-type SdeA, SdeA catalytic mutant SdeA H277A or SdeA EE/AA, truncated SdeA^1-972^ and SdeA^909-C^. **B** Confocal images showing the colocalization of SdeA (green) with ER protein Calnexin (red). COS7 cells were transfected with plasmids encoding GFP-tagged wild type SdeA or truncated mutant. Cells were cultured for 24 h after transfection, then fixed, permeabilized, and stained with Calnexin antibody and visualized using confocal microscope. Scale bars, 10 μm. **C** Confocal images showing the colocalization of SdeA (green) with Golgi protein GM130 (red). Cells were cultured for 24 h after transfection, then fixed, permeabilized, and ultimately stained with GM130 antibody and visualized using confocal microscope. Scale bars, 10 μm.

Previous structural and biochemical studies have revealed the structure of SdeA catalytic core, and the mechanism by which SdeA ubiquitinates substrates is well established [11–13]. However, the function of the carboxyl terminal (CT) coiled-coil region, remained unknown (Fig. 1A). Previous reports suggested that coiled-coil domains are required for membrane localization of many *Salmonella* type III effectors [16]. In view of that SdeA co-localizes with ER protein calnexin and ubiquitinates many ER proteins, such as RTN4 and FAM134B [15, 17], we hypothesized that CT domain of SdeA is responsible for its membrane association. To test this, we first investigated the ER localization of wild-type SdeA and truncated SdeA^1-972^ mutant lacking the last part of the C-terminal region (Fig. 1A). We observed that ectopically expressed SdeA colocalized with ER protein Calnexin in COS7 cells, consistently with a previous study [18]. However the truncated SdeA did not co-localize with Calnexin but showed a rather cytosolic distribution (Fig. 1B). In addition, we observed that part of SdeA was densely localized close to the nucleus in cells (Fig. 1B). Staining with the Golgi marker GM130 revealed that this part of SdeA colocalized with the Golgi apparatus, while the truncated mutant SdeA^1-972^ did not (Fig. 1C). We confirmed that the C-terminus region of SdeA is necessary to its Golgi localization by expressing the N-terminal-truncated SdeA^909-C^ in cells (Fig. 1C). This data suggests that C-terminal part of SdeA is critical for its ER as well as Golgi membrane localization.

### SdeA induces disruption of Golgi structure

**Fig. 2:**
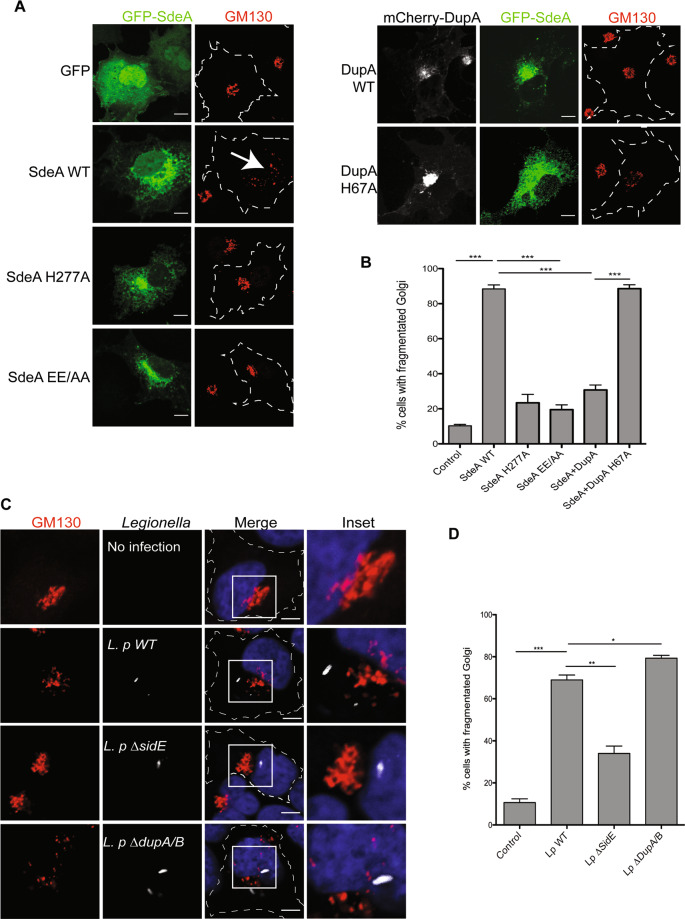
*Legionella* effector SdeA mediates Golgi fragmentation in cells. **A** Confocal images showing Golgi (red) fragmentation caused by exogenously expressed SdeA (green). GFP-tagged SdeA wild-type or catalytically defective mutants were expressed or co-expressed with DupA in COS7 cells. Cells were cultured for 24 h after transfection then fixed with 4% PFA. Scale bars, 10 μm. **B** Quantification of the percentage of cells with dispersed Golgi in (**A**). Data are shown as means ± SEM of more than 60 cells taken from three independent experiments. ***P < 0.001. **C** Confocal images showing Golgi fragmentation caused by *Legionella*. A549 cells were infected with wild-type or mutant *Legionella* as indicated. Cells were washed 3 times with PBS after 2 h infection to remove non-phagocytosed bacteria, then fixed with 4% PFA and stained with indicated antibodies. Scale bars, 5 μm. **D** Quantification of the percentage of cells with dispersed Golgi in (**C**) Data are shown as means ± SEM of more than 70 cells taken from three independent experiments. Data were analyzed with unpaired *t* test, ***P < 0.001, *P < 0.01, *P < 0.05.

To investigate whether Golgi localization of SdeA is critical for its ligase function, we co-expressed wild-type SdeA or the truncated mutant SdeA^1-972^ with its known Golgi associated substrate Rab33b [9]. Western blot analysis showed that the truncated form of SdeA could not ubiquitinate Rab33b even though it was able to ADP-ribosylate ubiquitin (Fig.S1A). This data suggests that the C-terminus region of SdeA is critical not only for its localization but also for its ability to ubiquitinate Golgi proteins. During our localization studies, we observed that expression of wild-type SdeA, but not the CT-truncated mutants, results in dispersed GM130 staining (Fig. 1C). This implicates an effect of SdeA activity on the structural organization of the Golgi apparatus. Indeed, we found that expression of PDE defective mutant (SdeA H277A) or mART defective mutant (SdeA EE/AA) did not exhibit significant impact on the structure of the Golgi (Fig. 2A, B). In addition, the effect of wild-type SdeA on the Golgi structure could be counteracted by expression of DupA, but not its catalytically mutant DupA H67A (Fig. 2A, B). These findings suggest that Golgi disruption observed in cells expressing SdeA is likely caused by the accumulation of ubiquitinated substrates. These observations are in agreement with previous study [19]. Similar result was also obtained in HeLa cells stained with both cis (GM130) and trans (TGN46) Golgi marker antibodies (Fig. S1B). In order to evaluate the physiological relevance of SdeA in triggering Golgi disruption, we infected human lung carcinoma A549 cells with either a wild-type *Legionella* strain, or mutant strains (*ΔsidEs* or *ΔdupA/B*). As expected, we observed a scattering of the Golgi apparatus in cells infected with wild-type but not *∆sidEs Legionella* or control cells. Infection by *Legionella* without DupA/B caused more dramatic dispersal of the Golgi, compared to the wild-type *Legionella* (Fig. 2C, D). To further dissect the modulation of Golgi by SdeA, we analyzed the Golgi morphology of the cells expressing SdeA with super-resolution microscopy. Data from DNA-PAINT super-resolution microscopy shows that the Golgi structure was damaged, however cis Golgi protein GM130 and trans Golgi protein Golgin97 were still colocalized in cells expressing SdeA (Fig. S1C). Moreover, SdeA expression did not change the level of the proteins required for Golgi structure maintenance (Fig. S1D). Taken all together, these data suggest that SdeA mediated PR-ubiquitination of host substrates induces disruption but not complete fragmentation of Golgi ribbon.

### In vitro and in vivo validation of PR-ubiquitination of Golgi substrates by SdeA

**Fig. 3:**
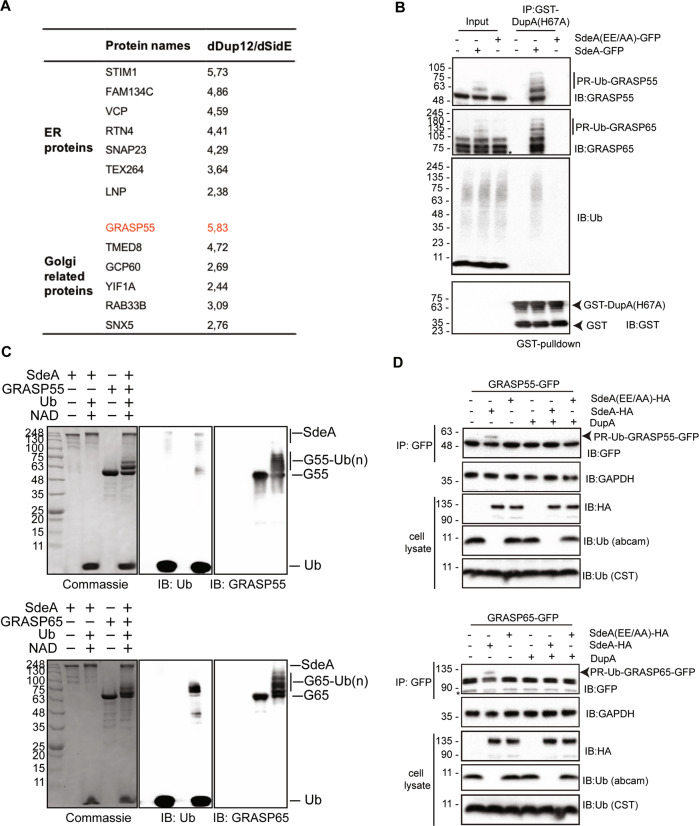
SdeA ubiquitinates Golgi tethering factor GRASP proteins. **A** Potential ER and Golgi protein substrates of SdeA identified by mass spectrometry. Values indicate intensity ratios between proteins enriched from samples infected with different *Legionella* strains (*ΔdupA/B* over *ΔsidE*). Among the substrate candidates, Golgi tethering factor GRASP55 (red) is one of the highly ubiquitinated proteins. **B** Modification of endogenous GRASP55 and GRASP65 by SdeA. Cell lysates from HEK293T cells expressing SdeA or SdeA EE/AA were incubated with GST-DupA (H67A) trapping mutant and blotted for GRASP55 and GRASP65. **C** GRASP55 and GRASP65 ubiquitination by SdeA in vitro. Purified GRASP55 or GRASP65 were incubated with SdeA in the presence of NAD^+^ and ubiquitin. Reaction products were separated with SDS-PAGE and then stained with Coomassie blue or blotted with antibodies against ubiquitin, GRASP55 or GRASP65. **D** Modification of overexpressed GRASP55 or GRASP65 by SdeA in cells. HEK293T cells were co-transfected with GFP-tagged GRASP55 or GRASP65 and wild type SdeA or indicated SdeA mutants, after 24 h cells were collected and lysed, GFP tagged proteins were isolated with GFP-trap beads, treated with purified DupA and separated with SDS-PAGE followed by blotting with antibody against GFP. For total cell lysate, the cell signaling technology (CST) antibody against ubiquitin was used to detect total ubiquitin, abcam antibody that does not detect ubiquitin modified by SdeA was used to indicate the expression and activity SdeA.

Using the PR-deubiquitinase DupA as a bait, we pulled-down over 180 potential host substrate proteins of SdeA [15]. Notably, Golgi proteins GRASP55 (gene name: GORASP2) and GCP60 (gene name: ACBD3) had the highest ratios among these putative Golgi protein substrates (Fig. 3A). Since GRASP55 plays important roles in the maintenance of the Golgi structure [20, 21], we hypothesized that SdeA modifies and inactivates Golgi proteins related to structure maintenance, thereby inducing Golgi disruption. GRASP65, which shares high sequence similarity with GRASP55, is localized to the *cis* Golgi and is also found in dispersed Golgi apparatus in cells expressing wild-type SdeA (Fig. S2A). To test this hypothesis, firstly we incubated GST-DupA H67A trapping mutant with cell lysate from cells expressing SdeA and blotted the pulldown product with antibodies against GRASP55 and GRASP65 to validate if SdeA modifies endogenous GRASP proteins. The blots show that SdeA expression indeed caused PR-ubiquitination of both GRASP55 and GRASP65 (Fig. 3B). In vitro ubiquitination assays were performed to further confirm the potential PR-ubiquitination of these two Golgi proteins by SdeA. We observed that SdeA is also able to modify both GRASP55 and GRASP65 in vitro (Fig. 3C). Furthermore, cellular expression of wild-type SdeA, but not inactive mART mutant, resulted in the appearance of ubiquitinated GRASP55 and GRASP65 in cells. This PR-ubiquitination was lost when pure DupA was added to immunoprecipitated GFP-tagged GRASP55 or GRASP65 in vitro (Fig. 3D). Similar observations were made for GCP60, where purified GCP60 from cells incubated with wild-type SdeA exhibited PR-ubiquitination (Fig. S2B). Such modification also appeared in cells when GCP60 was co-expressed with wild-type SdeA but not upon co-expression of SdeA EE/AA mutant (Fig. S2C). Along our hypothesis that SdeA is actively targeted to the Golgi, exogenous expression of CT-truncated SdeA mutants showed markedly reduced activity in modifying substrate GRASP55 (Fig. S2D), similar to the effect observed on PR-ubiquitination of Rab33b, indicating that Golgi localization of SdeA is important for substrate modification. Together, these results suggest that the PR-ubiquitination of Golgi tethering proteins GRASP55, GRASP65 and GCP60 by SdeA is a selective and functional part of the hijacking strategy of *Legionella*.

### *Legionella* infection causes PR-ubiquitination of GRASP55 and GRASP65

**Fig. 4:**
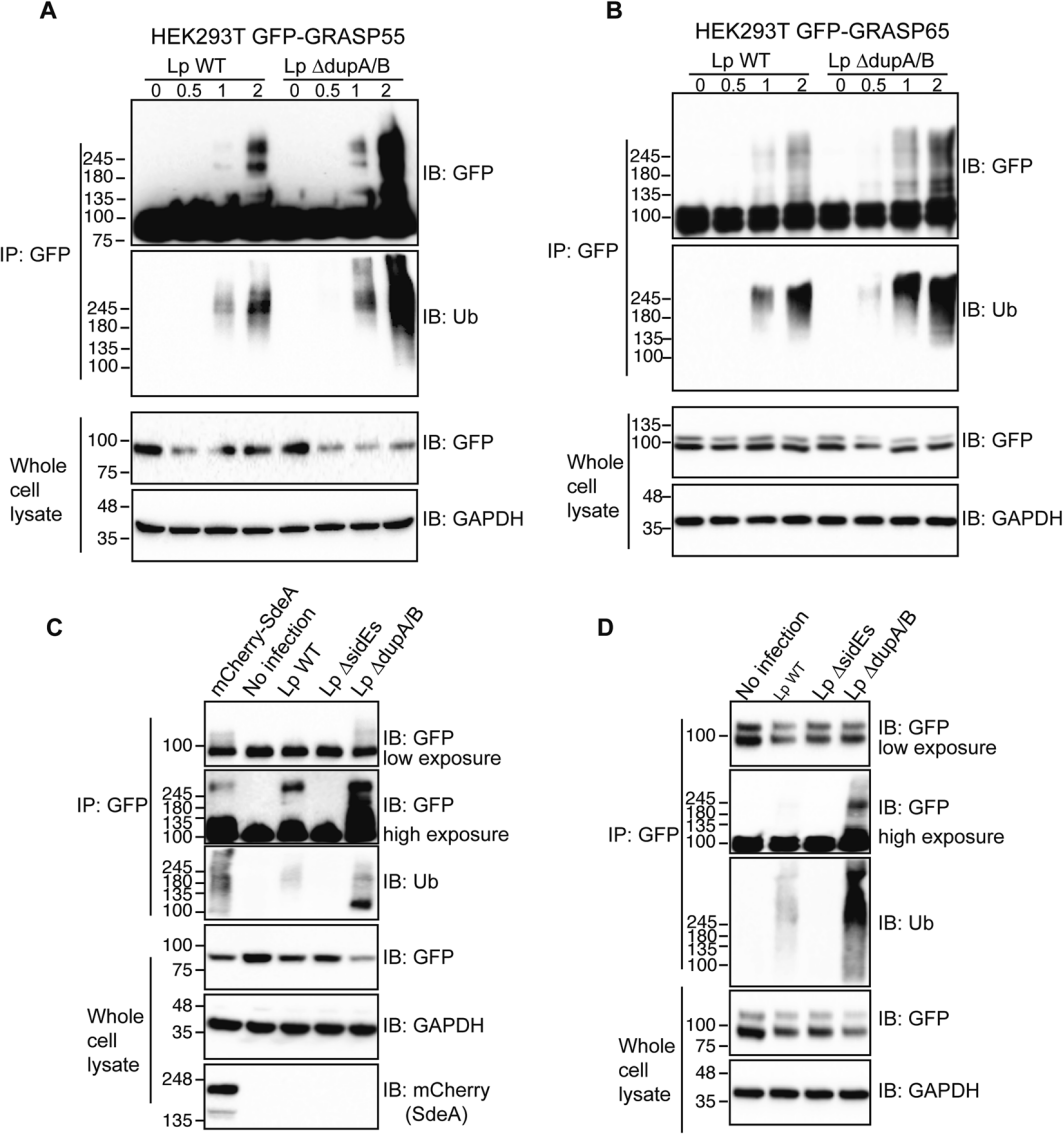
*Legionella* infection causes ubiquitination of GRASP proteins, which is dependent on SidE effectors. **A** Ubiquitination assay of GRASP55-GFP purified from HEK293T cells infected with *Legionella* strains. HEK293T cells were seeded in 6-well plate and co-transfected with plasmids encoding C-terminally GFP tagged GRASP55 and FcγRII then were infected for indicated times with *Legionella* bacteria opsonized by *Legionella* antibody. Cells were lysed with IP lysis buffer and purified GRASP55 proteins were separated by SDS-PAGE followed by blotting using anti-GFP and anti-ubiquitin antibodies. **B** Ubiquitination assay of GRASP65-GFP purified from HEK293T cells infected by *Legionella* wild type *or* Δ*dupA/B* mutant. **C** Ubiquitination assay of GRASP55-GFP purified from HEK293T cells infected with *Legionella* wild-type, Δ*sidEs or* Δ*dupA/B* strains. **D** Ubiquitination assay of GRASP65-GFP purified from HEK293T cells infected with *Legionella* wild-type, Δ*sidEs or* Δ*dupA/B* strains.

To check whether these Golgi proteins are PR-ubiquitinated upon *Legionella* infection, we immunoprecipitated GFP-tagged GRASP55 and GRASP65 from HEK293T cells infected with *Legionella* strains and analyzed them for PR-ubiquitination. The results showed that both GRASP55 and GRASP65 were ubiquitinated in a time-depending manner following *Legionella* infection (Fig. 4A, B). *Legionella* infection-induced GRASP55 and GRASP65 PR-ubiquitination was lost when cells were infected with *Legionella ∆sidEs*, and was increased in cells infected with the *Legionella ΔdupA/B* (Fig. 4C, D), thus, confirming that SidE family effectors are essential for PR-ubiquitination of host substrate proteins. Similar results were obtained for GCP60 (Fig. S3A). Moreover, DupA was able to remove the ubiquitination of GRASP55 and GRASP65 induced by *Legionella* infection (Fig. S3B, C). These data suggest that SdeA PR-ubiquitinates Golgi tethering proteins GRASP55 and GRASP65 during *Legionella* infection, further supporting our hypothesis that this modification has a directed function.

### SdeA ubiquitinates multiple serines of GRASP55 protein

**Fig. 5:**
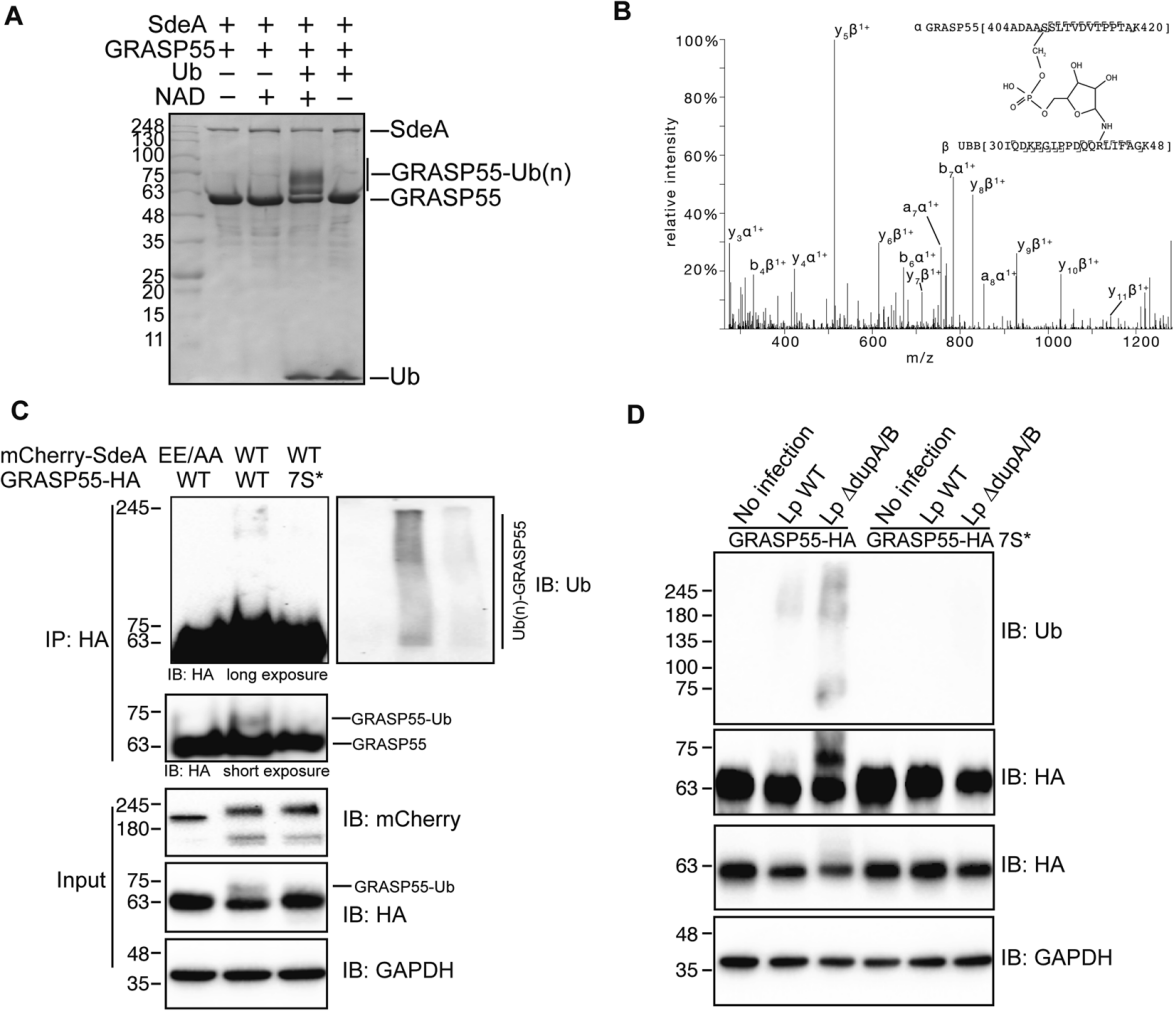
Identification of GRASP55 ubiquitination with mass spectrometry. **A** In vitro reaction of GRASP55 ubiquitination by SdeA for mass spectrometry analyses. In total, 20 μg purified GRASP55 was incubated with SdeA and ubiquitin in the present of NAD. 10% reaction products were separated with SDS-PAGE and then stained with Coomassie blue or blotted with with antibodies against ubiquitin, GRASP55 to check the ubiquitination, the rest samples were subjected to mass spectrometry analyses. **B** Spectrum of GRASP S408-ubiquitin cross-linked peptide. **C** Validation of of ubiquitination sites in GRASP55. C-terminally HA-tagged wild-type and GRASP55 mutant were co-expressed with SdeA in HEK293T cells. After 24 h the cells were lysed for HA immunoprecipitation. Purified GRASP55-HA proteins were separated with SDS-PAGE followed by blotting using anti-HA and anti-ubiquitin antibodies. **D** Ubiquitination assay of wild type GRASP55 and mutant in cells infected with *Legionella*.

Previous studies provided insights how SdeA targets and bridges Arg42 of Ub to serine residues of substrate proteins via a phosphoribosyl linker [9, 10]. To gain insight into the mechanism of activity regulation of GRASP proteins by PR-ubiquitination, we used mass spectrometry to identify modified residues on GRASP55 following in vitro ubiquitination by SdeA (Fig. 5A). Four modified serine residues were identified in GRASP55 (S3, S408, S409, S449) (Fig. 5B, Fig. S4). To further confirm these ubiquitination sites, we replaced seven serine residues (GRASP55 7 S*), including the identified serines and their adjacent serines, by either threonine (S3, S4, S449, S451) or alanine (S408, S409, S441). We observed that ubiquitination of GRASP55 in cells co-expressing SdeA was markedly decreased when the serines were replaced (Fig. 5C). Similarly, we confirmed that GRASP55 bearing the seven mutated serine residues can not be ubiquitinated when cells were infected with wild-type or *ΔdupA/B Legionella* strains (Fig. 5D). These results confirm that these identified serines are the primary targets.

### PR-ubiquitination disrupts GRASP interactions

**Fig. 6:**
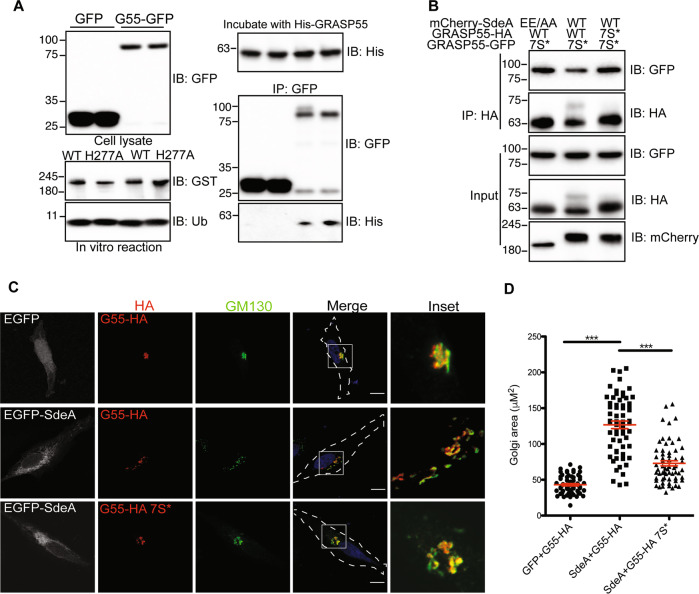
Serine ubiquitination impairs GRASP55 function. **A** Assay of the effect of PR-ubiquitination on GRASP55 dimerization in vitro. GRASP55-GFP purified from HEK293T cells were modified in vitro using SdeA and ubiquitin in the presence of NAD^+^, ubiquitinated GRASP55-GFP was then incubated with purified His-tagged GRASP55. Interaction between differently tagged GRASP55 proteins were analyzed with co-immunoprecipitation followed with western blotting. **B** Assay of the effect of PR-ubiquitination on GRASP55 dimerization in vivo. HA-tagged GRASP55 and GFP-tagged GRASP55 serine mutant were co-expressed with SdeA in HEK293T cells. Protein interaction between differently tagged GRASP55 were analyzed with co-IP and western blotting. **C** Confocal images showing that GRASP55 mutant is resistant to Golgi fragmentation caused by SdeA expression. Golgi areas of more than 60 cells from 3 replicates of each condition were measured with ImageJ software. Scale bars, 10 μm. **D** Data are shown as means ± SEM of more than 70 cells taken from three independent experiments. Data were analyzed with unpaired *t* test, ***P < 0.001.

Studies have shown that GRASP proteins function in the connection of Golgi stacks and thereby Golgi structure maintenance through self-interaction and interactions with Golgi matrix proteins [20, 22, 23]. Their activity can be regulated by post-translational modifications, for example, phosphorylation of serines within GRASP proteins was shown to result in Golgi fragmentation [24]. We hypothesized that PR-ubiquitination of serines in GRASP proteins may affect self-interactions that are necessary for the connection of the Golgi stacks. To test this, we firstly PR-ubiquitinated purified GRASP55-GFP in vitro and then incubated the modified GRASP55 with purified His-tagged GRASP55. Co-IP analyses showed that PR-ubiquitinated GRASP55 exhibited reduced self-interaction compared to unmodified GRASP55 (Fig. 6A). This effect could also be seen in cells when the HA-tagged wild type GRASP55 or GRASP55 7 S* serine mutant were co-expressed with GFP-tagged GRASP55 7 S* in the presence of SdeA. The capacity of PR-ubiquitinated wild-type HA-GRASP55 to self-interact with GFP-GRASP55 7 S* was decreased in comparison to SdeA resistant HA-GRASP55 7 S* (Fig. 6B). To analyze the functional impact of this observation on cells, we expressed wild-type GRASP55 or the GRASP55 7 S* serine mutant in *GRASP55/GRASP65* KO HeLa cells, then monitored the structural stability of the Golgi in cells co-expressing SdeA. As previously shown, double knockout of *GRASP55* and *GRASP65* induced dispersal of the Golgi [25] (Fig. S5). This phenotype could be rescued by ectopic expression of either wild-type GRASP55 or GRASP55 7 S* (Fig. S5), suggesting that serine mutations do not interfere with the function of GRASP55. Golgi disruption re-occurred when SdeA was concomitantly expressed with GRASP55 (Fig. 6C, D). However, the Golgi apparatus appeared less scattered when GRASP55 7 S* was expressed, indicating that the higher resistance of GRASP55 serine mutant to SdeA ubiquitination activity results in increased structural stability of the Golgi in cells expressing SdeA (Fig. 6C, D). This data indicates that SdeA-caused Golgi disruption is supposedly the result of the modification of GRASP proteins, disturbing the connection between Golgi stacks.

### *Legionella* containing vacuole does not recruit Golgi scatters

**Fig. 7:**
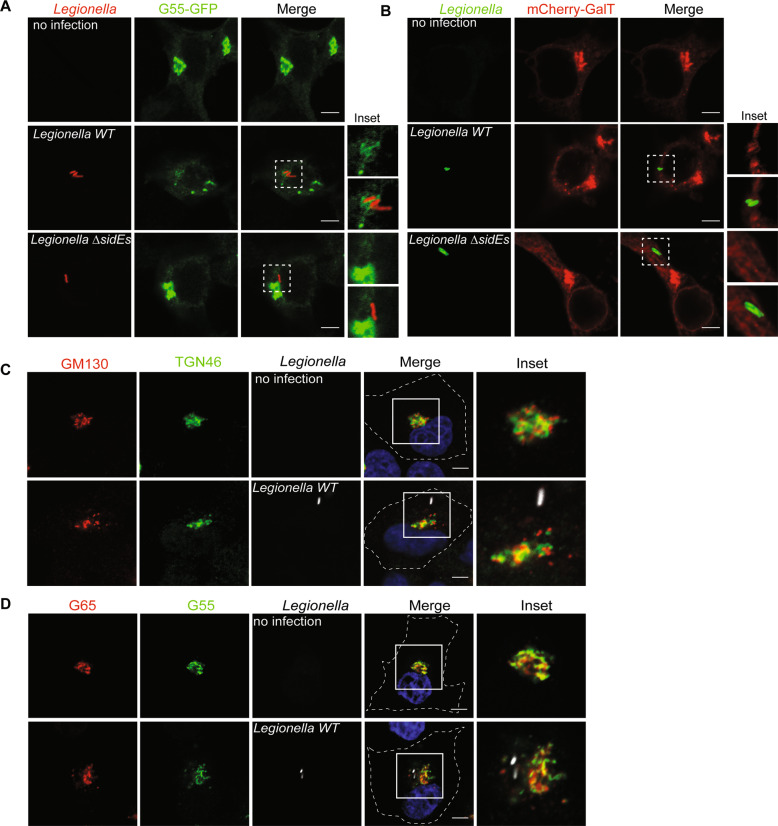
*Legionella* does not recruit fragmented Golgi. **A** Confocal images showing *Legionella* recruits overexpressed Golgi protein GRASP55. HEK293T cells transfected with plasmids encoding FCγRII and GFP-tagged GRASP55 were infected with indicated *Legionella* strains. Cells were washed 3 times with PBS after 2 h infection to remove un-endocytosed bacteria, then fixed with 4% PFA and stained with antibody against *Legionella*. Scale bars, 5 μm. **B** Confocal images showing *Legionella* recruits overexpressed Golgi marker GalT. Scale bars, 5 μm. **C** Confocal images showing *Legionella* does not recruit endogenous cis-Golgi protein GM130 or trans-Golgi protein TGN46. A549 cells were infected with Legionella expressing dsRed and stained with antibodies against GM130 and TGN46. Scale bars, 5 μm. **D** Confocal images showing *Legionella* does not recruit endogenous cis-Golgi protein GRASP65 or trans-Golgi protein GRASP55. A549 cells were infected with Legionella expressing dsRed and stained with antibodies against GRASP65 and GRASP55. Scale bars, 5 μm.

Intracellular pathogens tend to create a membrane surrounded niche for maturation, proliferation, and escape from defense mechanisms such as selective autophagy within the host cell. Along this line, *Chlamydia* infection induces Golgi fragmentation in order to generate Golgi ministacks for bacterial inclusions [26]. As for *Legionella*, *Legionella* containing vacuoles (LCVs) recruit ER membranes, thus converting the phagosome into a specific compartment that has features of ER [15, 17, 27]. We hypothesized that *Legionella* infection induces Golgi dispersal in order to facilitate the fusion of vesicles from the Golgi with LCV to enhance the formation of LCV and, ultimately, intracellular replication. To test this hypothesis, we infected HEK293T cells overexpressing GRASP55 or trans-Golgi marker GalT. The immunostaining showed that exogenous GRASP55 was recruited to LCV, however, our study recognized the fact that exogenously overexpressed GRASP55 and GalT were shown to be partially localized in ER, which can be remodeled and recruited to LCV during infection. The recruited GRASP55 could very well be derived from the ER, and not the fragmented Golgi (Fig. 7A, B). To further address whether *Legionella* recruits fragmented Golgi cargo, we infected A549 cells with *Legionella*, stained cells with antibodies against endogenous cis-Golgi protein GM130 or trans-Golgi protein TGN46. Immunostaining results suggested that neither cis-Golgi marker nor trans-Golgi accumulated on LCV (Fig. 7C, D). These data suggest that against our initial hypothesis *Legionella* does not disperse the Golgi simply to recruit Golgi-derived vesicles for the creation of LCVs, but that there must be another functional reasoning behind.

### Serine ubiquitination regulates secretory pathway in host cells

**Fig. 8:**
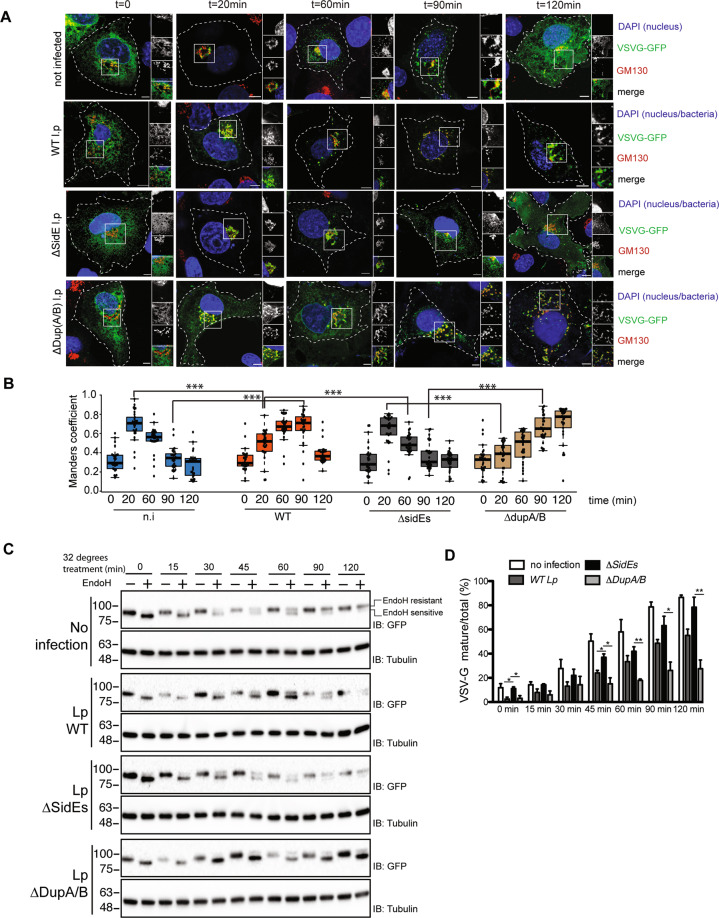
SdeA induced serine ubiquitination inhibits VSVG trafficking through Golgi membranes. **A** Confocal images showing the effect of SidE family effectors on VSVG trafficking during *Legionella* infection. A549 cells were transfected with VSV-G-GFP and cultured at 37 °C for 24 h to express the proteins before transferred to 40 °C. After 16 h incubation at 40 °C, cells were infected with *Legionella* for another 2 h then washed 3 times with PBS then moved to 32 °C for 0 min, 10 min, 20 min, 60 min, 90 min, 120 min to release VSVG from ER. A549 cells were fixed and VSVG trafficking was acquired with confocal microscopy after immunofluorescence staining. Scale bars, 5 μm. **B** Quantitative analysis of the effect of SidE family effectors on VSVG trafficking during *Legionella* infection. Colocalization between VSVG and GM130 was shown as Manders coefficient. Data represents 30 cells taken from 3 independent experiments. White boxes indicate insets which are split into red, green, blue channels and displayed on the right side of the image. Center lines show the medians; box limits indicate the 25th and 75th percentiles as determined by R software; whiskers extend 1.5 times the interquartile range from the 25th and 75th percentiles, outliers are represented by dots; data points are plotted as circles. **C** Western blotting analysis of the effect of SidE family effectors on VSVG trafficking during *Legionella* infection using EndoH. Upper bands indicate the EndoH resistant form (mature) and lower bands indicate the EndoH sensitive form of VSVG. **D** Quantification of (**C**) to indicate the effect of *Legionella* infection on the conversion of EndoH sensitive form to resistant form of VSVG upon 32 °C incubation. Data were analyzed with unpaired *t* test, ***P < 0.001, **P < 0.01, *P < 0.05.

In eukaryotic cells, the Golgi stack receives newly synthesized proteins from the ER, proteins then undergo modifications before being sorted via the trans-Golgi network. To investigate the effect of SdeA mediated Golgi disruption on Golgi function, we performed fluorescence recovery after photobleaching (FRAP) assay, the data indicates that the recovery of fluorescence after photobleaching of marked region is slower in SdeA expressing cells (Fig. S6). Based on this observation, we then asked that whether SdeA inhibits protein trafficking via the Golgi. Vesicular stomatitis virus glycoprotein (VSVG) is a transmembrane protein that has been widely used as a tool to monitor protein trafficking through the secretory pathway [28, 29]. To access the functionality of the Golgi apparatus upon *Legionella* infection, we used a VSVG-GFP tracker protein to follow its transit through the Golgi. Immunofluorescence analyses indicated that in control A549 cells or cells infected with *Legionella* SidEs deletion strain, VSVG reached its peak of accumulation in the Golgi after 20 min of incubation at 32 °C, and the colocalization index then gradually decreased as the protein is trafficked from the Golgi to secretory vesicles. This process was slower in cells infected with wild-type *Legionella* or *ΔdupA/B* mutant strain, where maximal colocalization of VSVG with the GM130 occurred at a later time point and was more prolonged, indicating lower efficiency of protein trafficking through the secretory pathway (Fig. 8A, B). This was further confirmed by monitoring the sensitivity of VSVG glycosylation to Endoglycosidase H (EndoH). EndoH is an enzyme that removes mannose rich ER resident protein but not complex forms of N-like oligosaccharides from glycoproteins that are present in the Golgi or post Golgi compartments, thus has been widely used to monitor protein trafficking through the Golgi [30, 31]. To specifically analyze the effect of PR-ubiquitination on VSVG trafficking, we infected HEK293T cells at 40 °C and collected cells lysates at different time points after incubation at 32 °C, before treating them with EndoH. Western blots showed that the band shift of VSVG from EndoH sensitive form to resistant form was inhibited in cells infected with wild type *Legionella* or *Legionella* DupA/B deletion strain, compared with that in control cells or cells infected with *Legionella* ∆sidE strain, indicating that VSVG trafficking through the Golgi was suppressed (Fig. 8C, D). These data further demonstrate that PR-ubiquitination caused by SidE effectors decelerates protein trafficking through the Golgi. This is also confirmed with a VSVG assay in cells expressing SdeA (Fig. S7A, B). Taken together, these data demonstrate that Golgi disruption caused by SidE effectors impairs protein secretory pathway.

## Discussion

To date, considerable effort has been focused on investigating the mechanism and substrates of novel PR-ubiquitination catalyzed by SidE family of *Legionella* effectors. However, the functional consequences of PR-ubiquitination in the regulation of cellular processes has been poorly understood. In this study, we investigated the modification of Golgi proteins catalyzed by SidE effectors and explored the consequences of PR-ubiquitination in regulating Golgi morphology and secretory pathway.

By conducting in vitro reactions and MS-based analyses, we identified several serine residues in GRASP55 potentially modified by SdeA. Notably, mutation of these serines did not completely abolish the ubiquitination signal from purified GRASP55, suggesting that alternative residues in GRASP55 could also be modified by SdeA. This finding is consistent to other known substrates like Rab33b, in which S154 has been identified as a ubiquitination site for SdeA, yet S154A mutation does not abrogate ubiquitination [10]. SdeA appears to modify substrate serine sites independent of specific structural motifs and serines in the flexible regions are prone to be modified as shown for Rab proteins [14].

GRASP proteins contain a conserved N-terminal GRASP domain that is used to localize the proteins to the Golgi as well as to tether other GRASP proteins through trans-dimerization, which can be regulated through phosphorylation of the C-terminal serine and proline rich (SPR) domain by mitotic kinases [24, 32]. Several serines in this C-terminal region of GRASP55 including S408, S409, S441, S449 identified to be PR-ubiquitinated here, have also been reported to be phosphorylated in previous studies [33, 34]. Phosphorylation mimics at these sites disrupt the homodimerization of GRASP, possibly through protein conformational changes [34, 35]. Based on the findings from a study using in vivo GRASP55/66 depletion, Grond et al. proposed that, instead of stacking the Golgi cisternae core, GRASP proteins function in linking of the rims of Golgi cisternae and the consequent connection of Golgi stacks [20]. The disruption of GRASP protein homodimerization by PR-ubiquitination at these sites may lead to disconnection of Golgi stacks. Of note, in our previous study, some proteins related to, such as AKAP12, EPB41, SLK, were identified as possible substrates of SdeA [15]. This may also affect the assembly of organelles including the Golgi. More studies will be needed in future to answer the question whether *Legionella* regulates cytoskeleton organization through SdeA mediated PR-ubiquitination of these cytoskeletal proteins.

Many pathogens have been characterized to require host organelles for their own intracellular survival. As for *Legionella*, numerous host proteins have been detected on the LCVs. Of note, PI(4)P decoration on LCV, which functions to recruit bacterial effectors during infection, was shown to be derived directly from the Golgi body of host cells [36]. However, our data in this study suggest that *Legionella* effectors disperse the Golgi but are not involved in the recruitment of Golgi components. This is in agreement with earlier studies, in which LCVs were purified from infected host cells and analyzed using proteomics approach, but Golgi proteins were rarely identified [37, 38]. During our ongoing study and preparation of this manuscript, Wan and colleagues reported Golgi fragmentation upon *Legionella* infection and the PR-ubiquitination of GRASP55. In their study it was shown that GRASP55 was recruited to LCV upon *Legionella* infection [39]. However, it should be noted that, unlike the endogenous GRASP55 protein that mainly localizes to the Golgi apparatus, overexpressed GRASP55 in their study was detected as largely localized to ER that could be recruited to LCV. The recruited GRASP55 could very well be derived from the ER, but not the dispersed Golgi. Recently, a study reported that PI(4)P-containing vesicles derived from Golgi are involved in mitochondria division [40]. Given the fact that mitochondrial dynamics is tightly modulated during *Legionella* infection [41], it is possible that *Legionella* SdeA affects mitochondria fission to facilitate bacterial replication. Further efforts will be needed to address the effect of PR-ubiquitination mediated Golgi disruption on mitochondria.

We have shown that PR-ubiquitination decelerates VSVG trafficking through the Golgi using microscopy and EndoH digestion assay. This finding is consistent with previous study showing that SdeA expression inhibits secretion of secreted embryonic alkaline phosphatase reporter (SEAP) [9].

Notably, multiple *Legionella* effectors have been suggested to regulate secretory pathways by yet unclear mechanisms [42, 43]. Identification of effectors involved in the regulation of the host secretory pathways will help us better understand both the bacterial pathogen and host cellular processes involved in infection, and thus further studies are needed.

Taken together, our study demonstrates that SdeA targets the Golgi and ubiquitinates Golgi tethering proteins GRASP55 and GRASP65, resulting in Golgi disruption and inhibition of secretory pathway. By revealing the biological consequences of PR-ubiquitination on Golgi proteins, our study provides a Golgi manipulation strategy, which *Legionella* utilizes to benefit bacterial infection. It will be interesting to study whether PR-ubiquitination confers additional versatile mechanisms to facilitate bacterial infection by verifying more substrates of SidE effectors in future.

## Materials and methods

### Antibodies and reagents

All reagents were from Sigma, Roche or Roth. The following antibodies were used: antibodies against HA (C29F4), GFP (sc-9996), GRASP65 (sc-374423), from Santa Cruz; ubiquitin (P4BD) and ubiquitin (ab7254) from Cell signaling and Abcam respectively; mCherry (ab125096), Tubulin (ab6046), Calnexin (ab22595), *Legionella* (ab20943) from abcam; GM130 (D6B1), GAPDH (D16H11) from Cell signaling; GM130 (610823) from BD for IF only; GRASP55 (10598-1-AP) from proteintech, TGN46 (AHP500) from Biorad. Monoclonal Anti-HA−Agarose antibody (HA-7) was purchased from Sigma.

### Cloning and mutagenesis

For protein expression in mammalian cells, GFP or mCherry tagged DupA, wild-type EGFP-SdeA and catalytically defective mutants SdeA H277A and SdeA EEAA were generated as described previously [10]. SdeA plasmids were digested with BamHI and XhoI, then inserted into mCherry-C1 vectors digested with BamHI and XhoI to generate N terminally mCherry tagged wild-type and mutated SdeA. Deletion of SdeA was designed according to the known structure and sequence prediction analyses. Truncated deletions SdeA^1-972^ and SdeA^909-C^ were amplified from full-length SdeA cDNA and digested with BamHI and XhoI. The digested DNA fragments were inserted into pEGFP-C1 vectors digested with BamHI and XhoI. GFP or HA tagged GRASP55 and GRASP65-GFP were generated by PCR from GRASP55 or GRASP65 cDNA and digested with XhoI and BamHI or HindIII and KpnI respectively, then inserted into pEGFP-N1 or pHA-N1 vector. For generation of the GRASP55 7 S* mutant, identified serines and adjacent serines S3, S4, S449, S451 were mutated to threonine to minimally effect the physio-chemical properties of these amino acids, in addition, S408, S409, S441 were mutated to alanine by site-directed mutagenesis. For protein expression in *E. coli*, SdeA was amplified from SdeA cDNA and digested with BamHI and XhoI. The digested DNA fragments were inserted into pGEX-6p-1 vector digested with BamHI and XhoI. GRASP55 and GRASP65 cDNA were amplified from mammalian vector and digested with NdeI and BamHI and cloned into pET15b and pGEX-6p-1 vector respectively. Serine to threonine or alanine mutations were generated by site-directed mutagenesis.

### Cell lines culture and transfection

HEK293T, A549, COS7 cells were purchased from ATCC. Cells were cultured in high glucose Dulbecco’s Modified Eagles Medium (DMEM) supplemented with 10% fetal bovine serum (FBS), 100 U/mL penicilin and 100 mg/mL streptomycin at 37 °C, 5% CO_2_ in a humidified incubator. Transfection was performed using polyethyleneimine (PEI) reagent or Genejuice (Merck).

### *Legionella* culture and infection

*Legionella* strains were obtained from Dr. Zhao-Qing Luo lab (Purdue University). Cells were streaked and cultured at 37 °C on N-(2-acetamido)−2-aminoethanesulfonic acid (ACES)-buffered charcoal-yeast extract (BCYE) agar plates for 3 days, followed by inoculation and growth for 20 h in 3 mL CYE liquid media. Post-exponential *Legionella* with OD_600_ between 3.6-3.8 were used to infect A549 or HEK293T cells. HEK293T cells were transfected with FCγRII and GRASP55-GFP or GRASP65-GFP for 24 h. Indicated *Legionella* strains were opsonized with antibody against *Legionella* (1: 500) at 37 °C for 30 min before infection. The HEK293T cells were infected with different *Legionella* strains at an MOI of 2 (for confocal imaging), or 10 (for Western blot) for the indicated time.

### SdeA mediated PR-ubiquitination reaction

SdeA mediated PR-Serine ubiquitination in vitro reaction was done as previously described [13]. Briefly, 5 μM GRASP proteins were incubated with 1 μM of SdeA and 25 of μM ubiquitin in the presence of 200 μM of NAD^+^ in 40 μL of reaction buffer (50 mM NaCl and 50 mM Tris, pH 7.5) for 1 h at 37 °C. Deubiquitination assay were performed by incubating PR-ubiquitinated proteins with 1 μg of GST-DupA at 37 °C for 1 h in reaction buffer (150 mM NaCl, 50 mM Tris-HCl pH 7.5). The reaction products were analyzed by SDS-PAGE with Coomassie staining or western blotting using antibodies against GST (cell signaling technology), His (Roche), GRASP55 (Proteintech), GRASP65 (Sino biotech.), Ub (ab7254 from Abcam, 39365 from Cell signaling technology). To assess the PR-ubiquitination of GRASP55 and GRASP65 in cells, plasmids for expression of GRASPs-GFP, GFP-SdeA or mCherry-SdeA, were co-transfected into HEK293T cells, cells were then cultured at 37 °C for 24 h. Whole cell lysates were subjected to immunoprecipitation with GFP-trap beads and the products or the whole cell lysates were separated with SDS-PAGE and blotted with antibodies against GFP or GRASP proteins.

### Western blotting and immunoprecipitation

Cell lysates or immunoprecipitated proteins were mixed with SDS sample buffer, heated at 95 °C for 5 min, centrifuged, and separated by Tris-Glycine SDS-PAGE, and transferred to PVDF membrane (Millipore) at cold room. Blots were blocked with 5% nonfat milk for 1 h at room temperature and incubated with primary antibodies overnight at cold room or 2 h at room temperature and washed with TBST (0.1% Tween 20 in TBS) three times. The blots were further incubated with secondary antibodies for 1 h at room temperature and washed 3 times with TBST. The blots were incubated with ECL reagents (advansta), and chemiluminescence was acquired with the Bio-Rad ChemiDoc system. For immunoprecipitation, HEK293T cells expressing GFP or HA-tagged proteins were lysed with mild immunoprecipitation buffer containing 150 mM NaCl, 50 mM Tris-HCl, pH 7.5, 0.5% NP40, 1 mM PMSF, protease inhibitor cocktail (Sigma Aldrich), mixed with 10 μL GFP-trap or HA antibody conjugated agarose, and incubated for 4 h in cold room with end to end rotation. Beads were washed 3 times in IP buffer containing 500 mM NaCl. Proteins were eluted by resuspending with 2X SDS sample buffer followed by boiling for 5 min at 95 °C. Samples were then submitted to western blotting analysis.

### Protein expression and purification

GRASP55 and GRASP65 cDNA were cloned into p15b and pGEX-6p-1 vector respectively. Full-length SdeA was cloned into pGEX-6P-1 vector. *E. coli* competent cells (NEB T7 express) were transformed with plasmid, colonies were inoculated and cultured in LB medium overnight at 37 °C, The next day 5 mL culture was transferred to 1 L flask for further culture at 37 °C until the OD_600_ reaches to 0.6–0.8. Protein expression was induced by adding 0.5 mM IPTG and cells were further cultured overnight at 18 °C. The cells were harvested and the cell pellet was resuspended in lysis buffer (300 mM NaCl, 50 mM Tris-HCl pH 7.5) followed with sonication and centrifuged at 13,000 rpm to clarify the supernatant. Clarified lysates were then incubated with TALON beads or glutathione-S-Sepharose pre-equilibrated with washing buffer. Once eluted, proteins were further concentrated with filters and then purified by anion exchange chromatography on HitrapQ (GE Healthcare) and collected fractions were further loaded onto size exclusion column (Superdex 75 16/60, GE Healthcare). Proteins were concentrated and used for in vitro reaction.

### Identification of PR-ubiquitination serine sites on GRASP55

His-GRASP55 were purified from *E. coli* and PR-ubiquitinated by SdeA in vitro. Samples were prepared as previously described [10, 13]. Briefly, added urea buffer containing 8 M urea, 0.1 M Tris, pH 7.5 to the reaction mixture to a final volume of 200 µL, the reactions were then transferred to 30 kDa filter (Amicon Ultra, 0.5 mL, Merck) and washed 3 times with 200 µL of urea buffer by centrifugation to remove free ubiquitin. Proteins were washed 2 times with 50 mM ABC, pH 7.5, then digested with trypsin in 50 mM ABC pH 7.5 at trypsin to protein ratio 1:50 for 6 h and subsequently desalted by C18 and analyzed by LC MS/MS.

### Data quantification

Data shown in Figs. 2B, 2D, 6D, 8B, 8D were analyzed with GraphPad Prism 5.0. Three independent experiments were performed, p values were determined using unpaired two-tailed *t* test, ***, **, * and ns represent *p* < 0.0001, *p* < 0.01, *p* < 0.05 and not significant respectively. For Fig. 2B and 2D, more than 70 SdeA transfected or *Legionella* infected cells were examined from 3 replicates of each condition. Values of percentage of cells with fragmented Golgi were input into GraphPad Prism, and analyzed. For Fig. 6D, Golgi areas of more than 60 cells from 3 replicates of each condition were measured with ImageJ software. For Fig. 8B, data represents 30 cells taken from 3 independent experiments. For Fig. 8D, gray values of VSVG bands shown as Fig. 8C from 3 replicates were measured with ImageJ.

### VSVG trafficking assay

HEK293T or A549 cells were co-transfected with VSVG-GFP and FcγRII or transfected with VSVG-GFP respectively and cultured at 37 °C for 24 h to express the proteins before being transferred to 40 °C. After 16 h incubation at 40 °C, cycloheximide was added into medium to inhibit further protein synthesis, after 2 h treatment cells were infected with *Legionella* for another 2 h then washed 3 times with PBS and cultured with fresh medium at 32 °C to remove the bacteria outside host cells, and then moved to 32 °C for different time points to release VSVG from ER. A549 cells were fixed and VSVG trafficking was acquired with confocal microscopy after immunofluorescence staining. DAPI marks nucleus and cytosolic bacteria. For calculating Manders coefficient in FIJI, ROIs of 30 µm^2^ are chosen from the perinuclear region containing the Golgi marked by GM130. Manders coefficient is calculated using Coloc2 plugin in FIJI and denotes fraction of VSVG-GFP pixels that is positive for GM130. For EndoH cleavage assay, HEK293T cells were lysed with lysis buffer containing 1% SDS, 50 mM Tris, pH 8.0. Benzonase was added to reduce the viscosity caused by released DNA. Cell lysates were mixed with denaturing buffer then boiled for 10 min at 95 °C. Denatured proteins were incubated with EndoH for 3 h at 37 °C to cleave the EndoH sensitive form of glycosylation, final products were separated with SDS-PAGE and the EndoH-caused band shift was analyzed by blotting GFP.

### Immunofluorescence

HEK293T, COS7, or A549 cells were seeded on a coverslip in 12-well plates and cultured in CO_2_ incubator. Next day cells were transfected with plasmids encoding SdeA. The immunostaining was performed following the protocol previously described [10]. Briefly, cells were washed once with PBS, pH 7.4, and fixed with 4% paraformaldehyde (PFA) in PBS for 10 min at room temperature. Cells were washed again with PBS 2 times, then permeabilized with 0.1% saponin in PBS for 10 min, and blocked with blocking buffer containing 0.1% saponin and 2% BSA in PBS for 1 h at room temperature. Cells were stained with antibodies diluted in blocking buffer overnight at 4 °C and washed with PBS three times next day. Cells were further incubated with Alexa Flour dyes-conjugated secondary antibodies for 1 h at room temperature in the dark and washed with PBS and incubated with DAPI in PBS, followed with further 2 times washing with PBS. Confocal imaging was performed using the Zeiss LSM780 microscope system. Images were analyzed with Fiji software.

### DNA-PAINT super-resolution Imaging

COS7 cells were fixed with prewarmed (37 °C) 4% methanol-free formaldehyde (Sigma-Aldrich, Germany) in PBS for 10 min followed by three washing steps with PBS. Cells were quenched with 0.1% NaBH_4_ (Sigma Aldrich, Germany) for 7 min in PBS and washed thrice with PBS. Fixed cells were permeabilized and blocked in permeabilization/blocking-buffer, followed by incubation of primary antibodies against GM130 and Golgin 97 in permeabilization/blocking buffer for 90 min. Washed cells were then incubated with DNA-labeled secondary antibodies for 1 h and washed again. Finally, samples were post-fixed using 4% methanol-free formaldehyde for 10 min at room-temperature followed by three washing steps with PBS. For sequential DNA-PAINT imaging, 125 nm gold-beads (Nanopartz, USA) were used as fiducial markers. Exchange DNA-PAINT measurements were performed at the N-STORM super-resolution microscopy system (Nikon, Japan) equipped with an oil immersion objective (Apo, 100x, NA 1.49) and an EMCCD camera (DU-897U-CS0-#BV, Andor Technology, Ireland).

## Supplementary information

Figure S1

Figure S2

Figure S3

Figure S4

Figure S5

Figure S6

Figure S7

Supplemental material
