# Supplementary figures and images for: Serine-ubiquitination regulates Golgi morphology and the secretory pathway upon Legionella infection
Source: Cell Death Differ. 2021 Jul 20;28(10):2957–69. doi: 10.1038/s41418-021-00830-y (PMC8481228; doi:10.1038/s41418-021-00830-y)

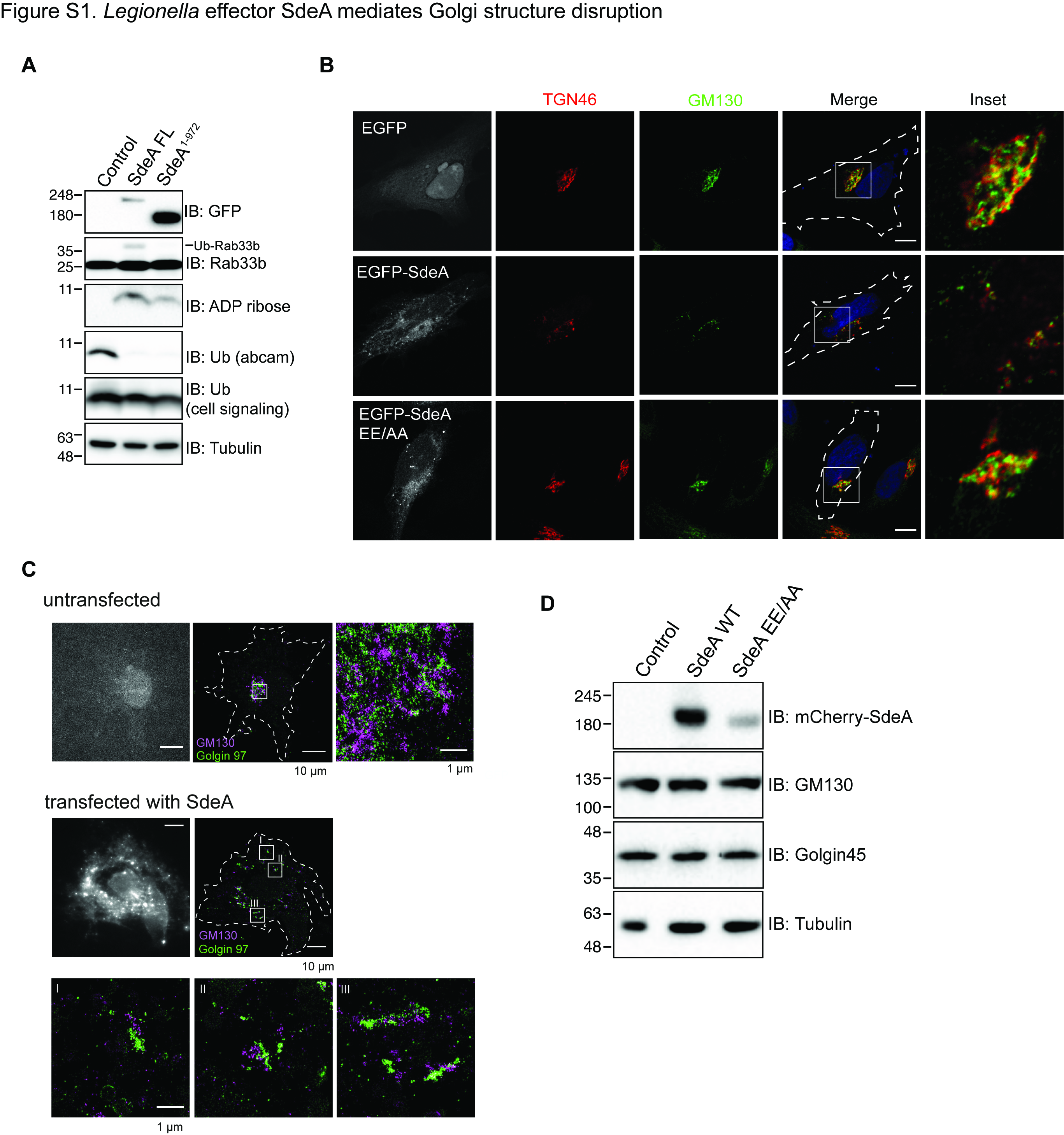

Supplement: Supplementary file 1 — Figure S1 [file 41418_2021_830_MOESM1_ESM.tif]

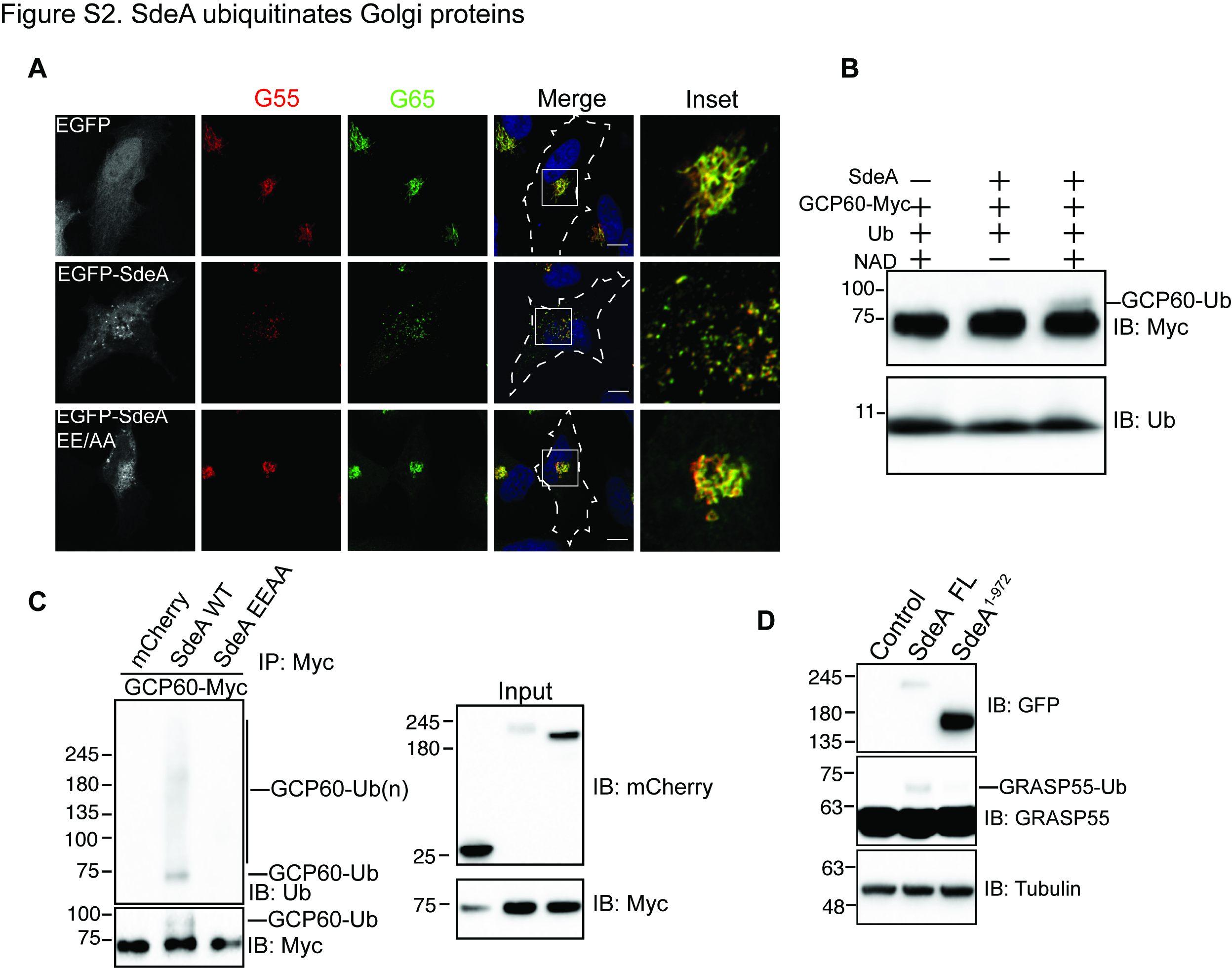

Supplement: Supplementary file 2 — Figure S2 [file 41418_2021_830_MOESM2_ESM.tif]

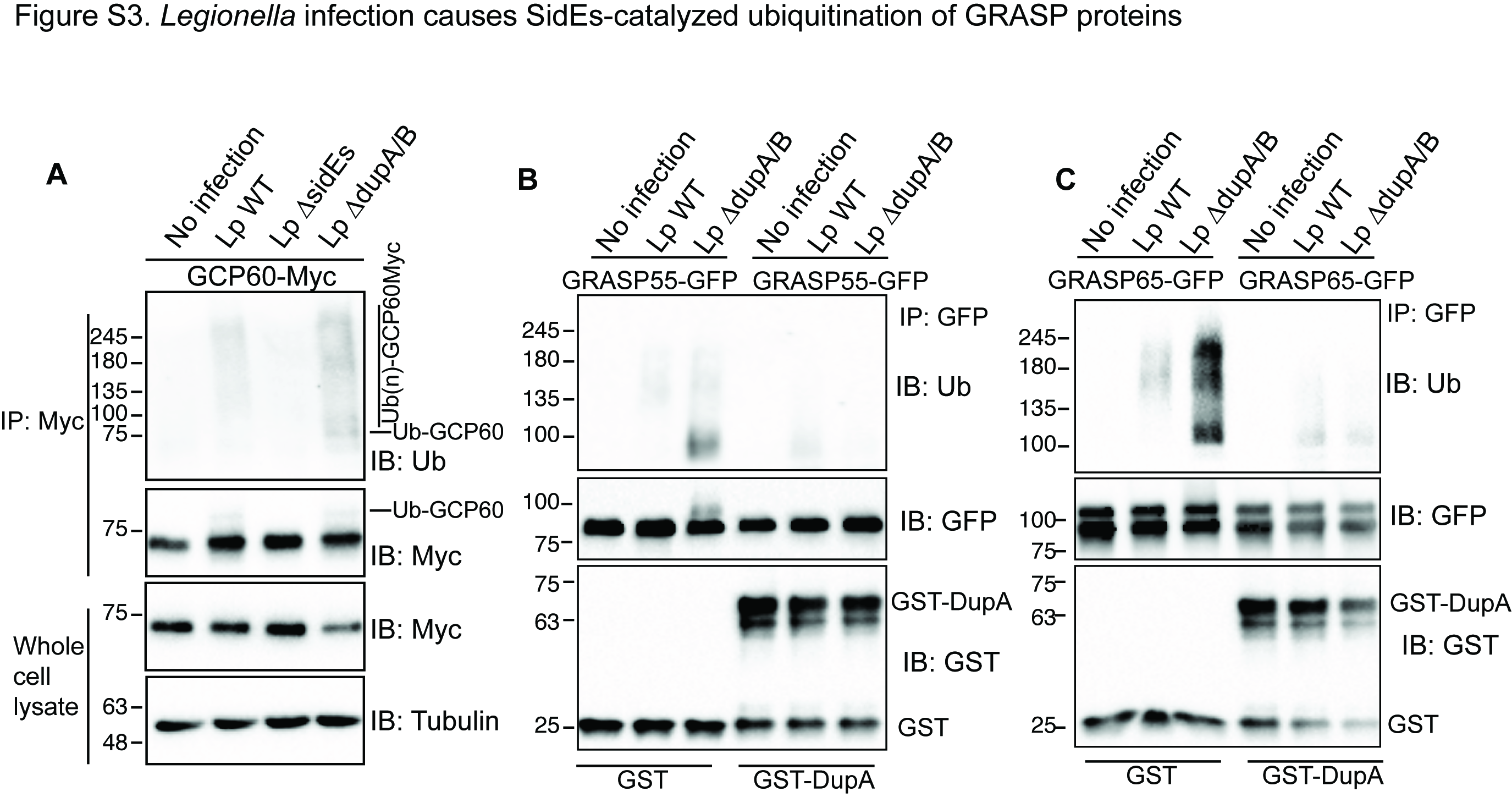

Supplement: Supplementary file 3 — Figure S3 [file 41418_2021_830_MOESM3_ESM.tif]

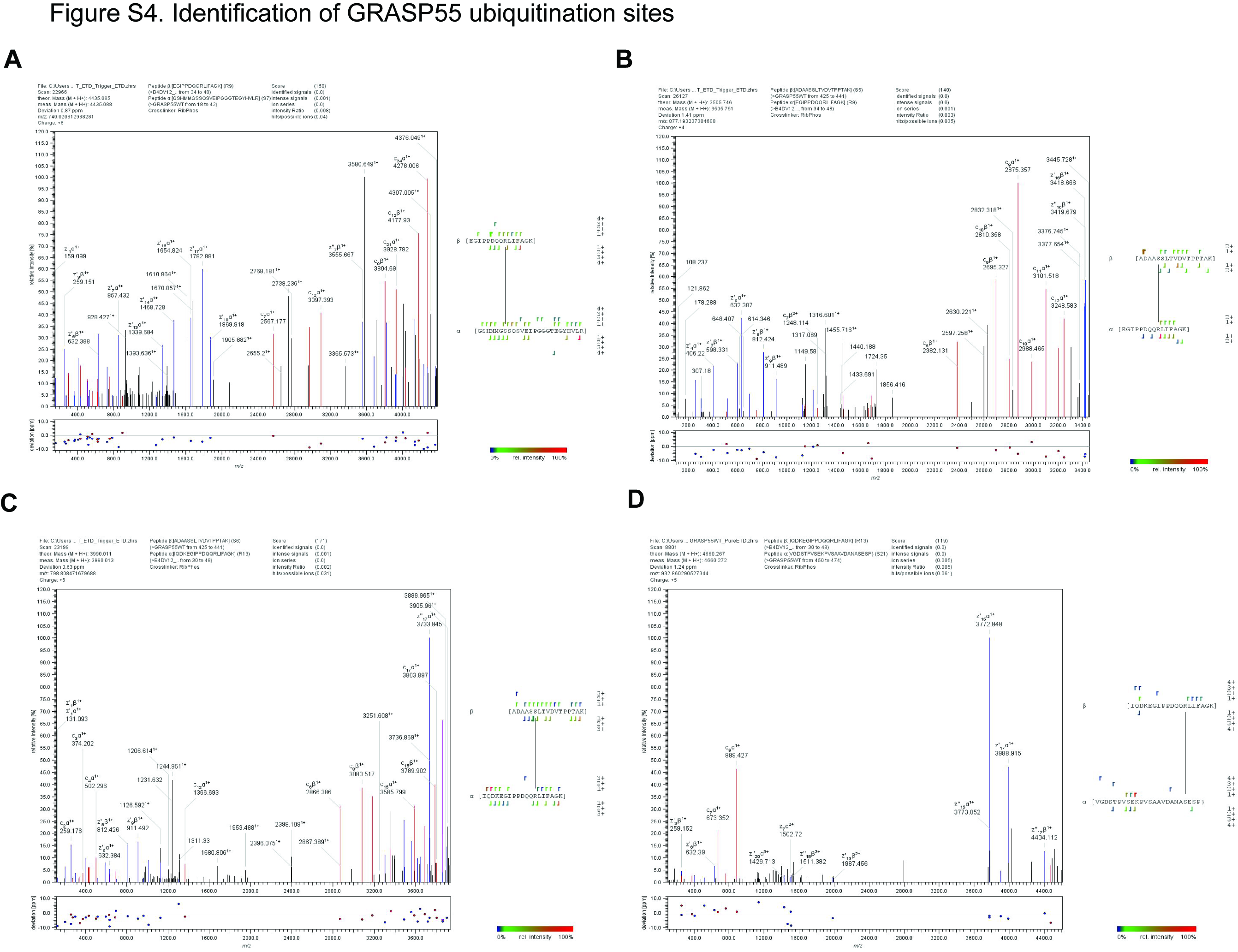

Supplement: Supplementary file 4 — Figure S4 [file 41418_2021_830_MOESM4_ESM.tif]

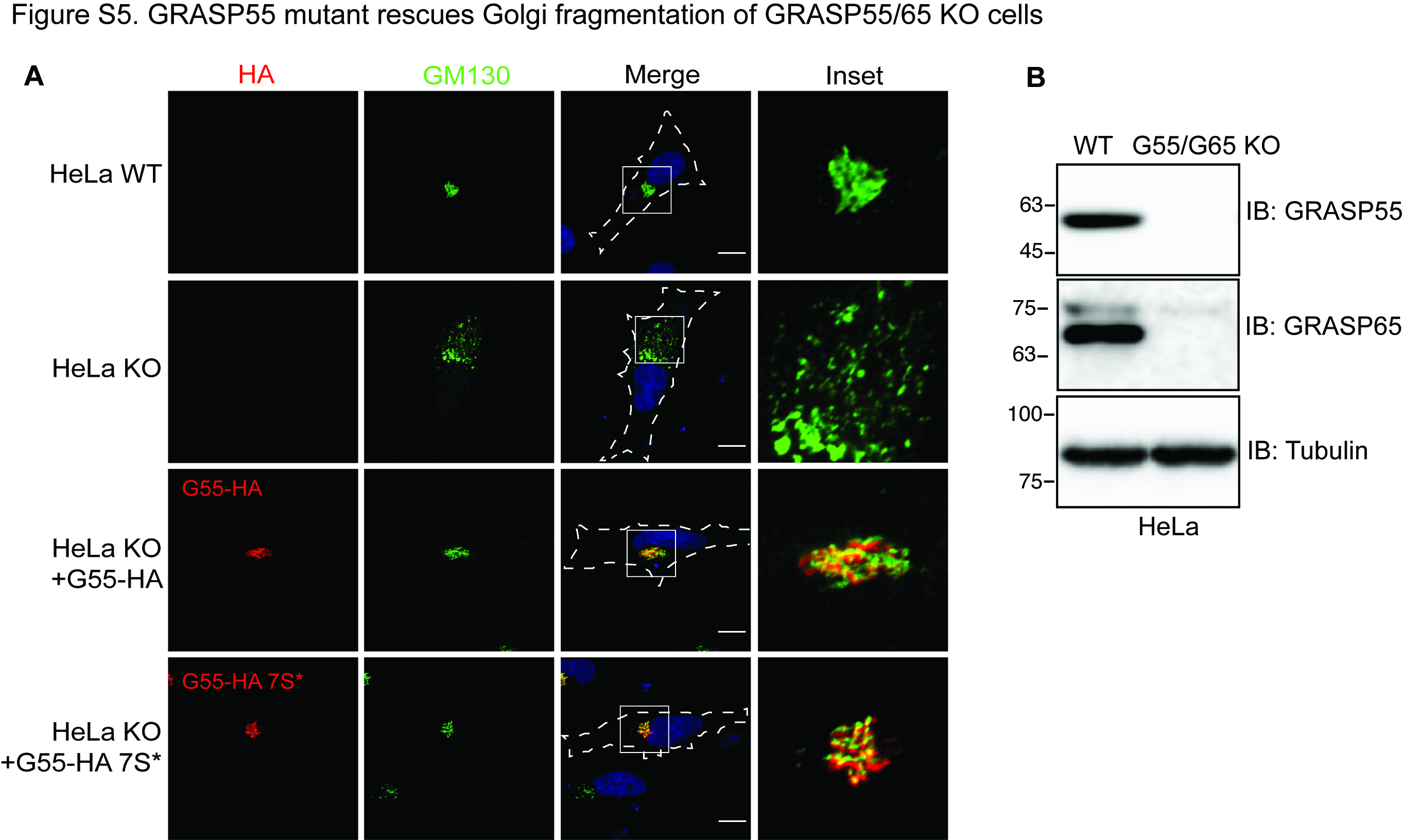

Supplement: Supplementary file 5 — Figure S5 [file 41418_2021_830_MOESM5_ESM.tif]

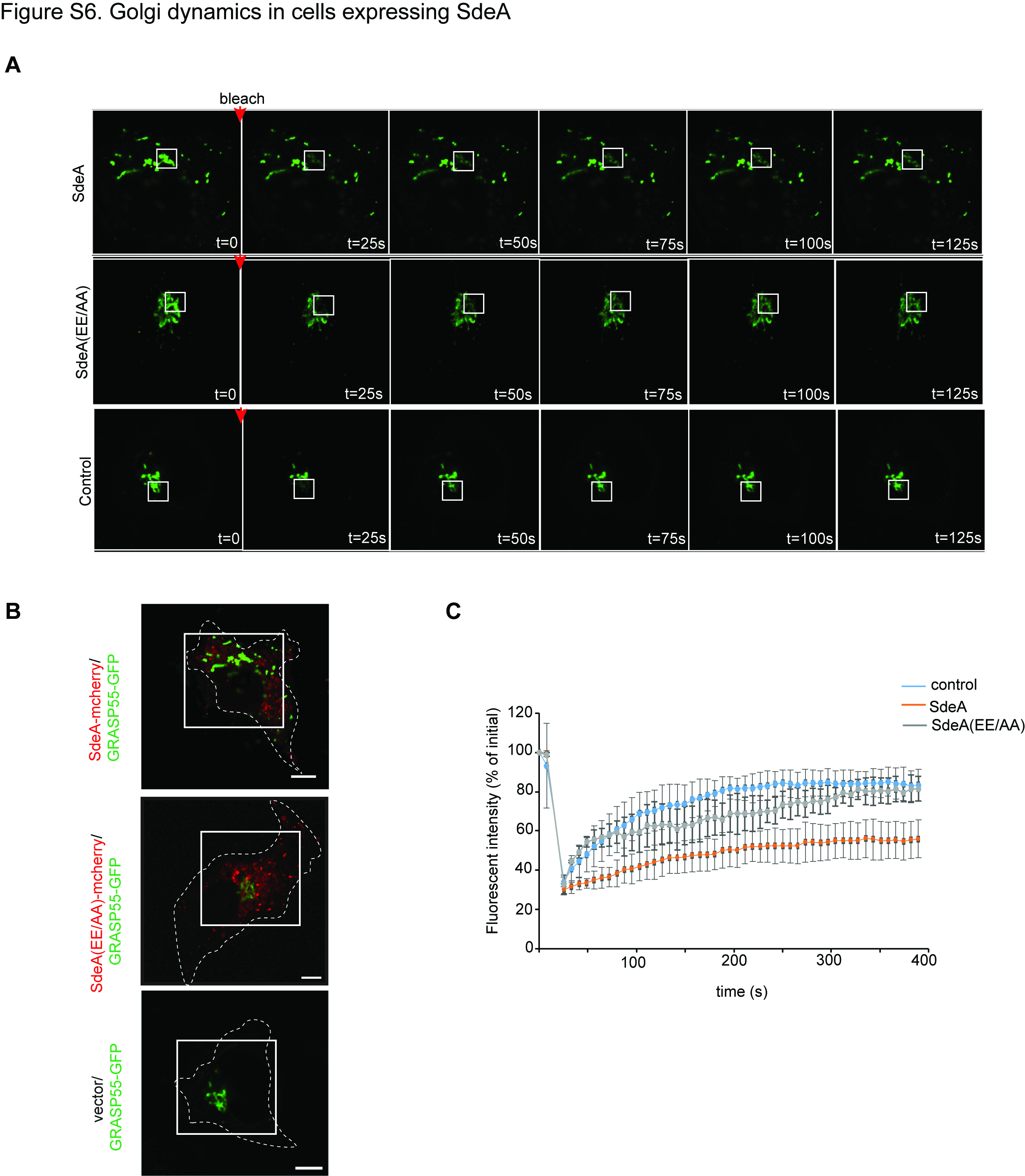

Supplement: Supplementary file 6 — Figure S6 [file 41418_2021_830_MOESM6_ESM.tif]

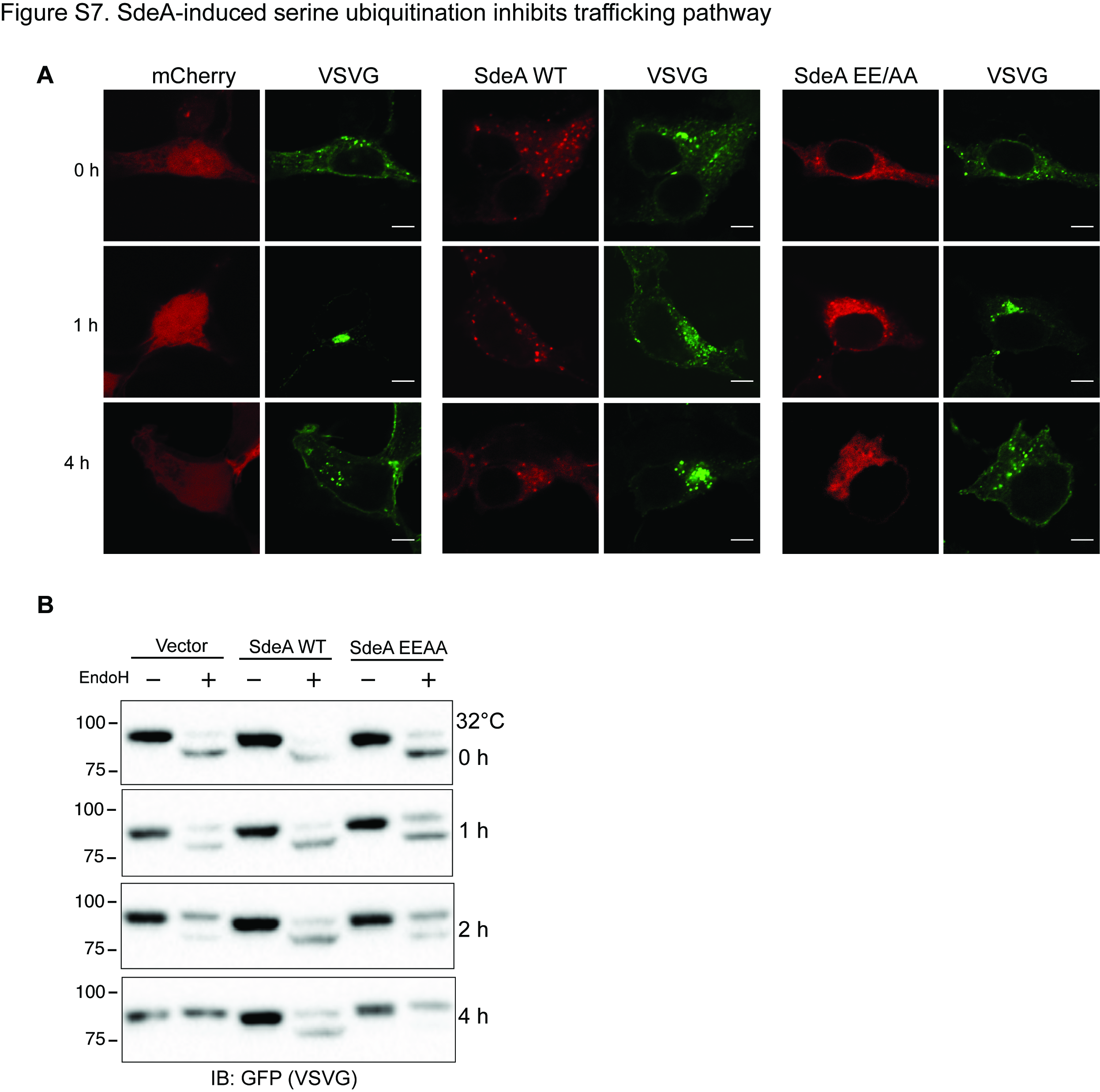

Supplement: Supplementary file 7 — Figure S7 [file 41418_2021_830_MOESM7_ESM.tif]
