# Supplementary material for: Serine-ubiquitination regulates Golgi morphology and the secretory pathway upon Legionella infection
Source: Cell Death Differ. 2021 Jul 20;28(10):2957–69. doi: 10.1038/s41418-021-00830-y (PMC8481228; doi:10.1038/s41418-021-00830-y)
Supplement: Supplementary file 8 — Supplemental material [file 41418_2021_830_MOESM8_ESM.docx]

**Supplementary information**

**Figure legends**

**Figure S1**

(**A**) Western blot analysis of modification of Golgi protein substrate by wild-type GFP-tagged SdeA or SdeA^1-972^ missing membrane targeting region. HEK293T cells were transfected with full-length or truncated SdeA, cells were lysed and blotted after 24 hours transfection. (**B**) Confocal images showing SdeA expression fragments the Golgi in HeLa cells. GFP-tagged SdeA wild type or catalytically defective mutants were expressed in HeLa cells. Cells were cultured for 24 hours after transfection then fixed with 4% PFA, after permeabilization, cells were stained with antibodies against cis-Golgi and trans-Golgi markers GM130 and TGN46. Scale bars, 10 μm. (**C**) DNA-PAINT super-resolution microscopy images of COS7 cells expressing SdeA. Fixed cells were incubated with primary antibodies against cis-Golgi and trans-Golgi markers GM130 and Golgin97, followed by incubation with secondary antibodies labeled with short oligonucleotide sequences. Data acquisition was performed with the N-STORM super-resolution microscopy system. (**D**) Western blot analysis of Golgi proteins GM130 and Golgin45 in cells expressing SdeA.

**Figure S2. SdeA ubiquitinates Golgi tethering proteins**

(**A**) Confocal images showing Golgi localization of endogenous GRASP55 and GRASP65. Scale bars, 10 μm. (**B**) GCP60 ubiquitination by SdeA *in vitro*. Purified Myc-tagged GCP60 was incubated with SdeA in the present of NAD^+^ and ubiquitin. Reaction products were blotted with antibodies against ubiquitin or Myc. (**C**) Modification of GCP60 by exogenous SdeA in cells. HEK293T cells were co-transfected with GCP60 and wild type SdeA or SdeA mutant, after 24 hours cells were collected and lysed, followed with Myc-IP. IP products were washed and separated with SDS-PAGE and blotted with antibody. (**D**) Modification of Golgi substrate GRASP55 by wild-type SdeA or SdeA^1-972^ missing membrane targeting region.

**Figure S3. *Legionella* infection causes ubiquitination of Golgi proteins, which is dependent of SidE family proteins**

(**A**) Ubiquitination assay of GCP60-Myc purified from HEK293T cells infected with *Legionella* strains. (**B**) Cleavage assay of PR-ubiquitination of GRASP55 with DupA. (**C**) Cleavage assay of PR-ubiquitination of GRASP65 with DupA.

**Figure S4.** **High resolution ETD spectrum of ubiquitin cross** (**A**) High resolution ETD spectrum of ubiquitin cross linked Serine 3 of GRASP55. (**B**) High resolution ETD spectrum of ubiquitin cross linked Serine 408 of GRASP55. (**C**) High resolution ETD spectrum of ubiquitin cross linked Serine 409 of GRASP55. (**D**) High resolution ETD spectrum of ubiquitin cross linked Serine 449 of GRASP55.

**Figure S5. GRASP55 mutant rescues Golgi fragmentation of GRASP55/65 KO cells**

(**A**) Confocal images showing exogenously expressed wild-type GRASP55-HA and mutant rescue Golgi fragmentation caused by GRASP55/GRASP65 knockout. Scale bars, 10 μm. (**B**) Western blotting of cell lysates from wild-type and G55/G65 knockout HeLa cell lines. Knockout of G55 and G65 were validated with antibodies against G55, G65 respectively.

**Figure S6. Golgi dynamics in cells expressing SdeA**

(**A**) FRAP (fluorescence recovery after photobleaching) experiment showing Golgi dynamics in SdeA expressing cells. Golgi in live control or cells expressing SdeA (**B**) were photobleached at ROIs followed by measurement of fluorescence of ROIs over time. Scale bars, 5 μm. (**C**) Recovery curve of fluorescence after photobleaching of marked ROIs.

**Figure S7. SdeA expression in cells impairs protein trafficking**

(**A**) Analysis of VSVG trafficking in HEK293T cells expressing SdeA. Cells grown in 40 °C were moved to 32 °C for indicated time points. Scale bars, 5 μm. (**B**) Analysis of VSVG trafficking in HEK293T cells expressing SdeA using EndoH. Cells grown in 40 °C were moved to 32 °C for indicated time points. Cell lysates were probed with GFP antibody.
